# Multi‐omic strategies applied to the study of pharmacoresistance in mesial temporal lobe epilepsy

**DOI:** 10.1002/epi4.12536

**Published:** 2021-10-18

**Authors:** Estela M. Bruxel, Amanda M. do Canto, Danielle C. F. Bruno, Jaqueline C. Geraldis, Iscia Lopes‐Cendes

**Affiliations:** ^1^ Departments of Translational Medicine School of Medical Sciences University of Campinas (UNICAMP) Campinas Brazil; ^2^ Brazilian Institute of Neuroscience and Neurotechnology (BRAINN) Campinas Brazil

**Keywords:** complex inheritance, epigenomics, metabolomics, multifactorial inheritance, pharmacogenomics, proteomics, transcriptomics

## Abstract

Mesial temporal lobe epilepsy (MTLE) is the most common type of focal epilepsy in adults, and hippocampal sclerosis (HS) is a frequent histopathological feature in patients with MTLE. Pharmacoresistance is present in at least one‐third of patients with MTLE with HS (MTLE+HS). Several hypotheses have been proposed to explain the mechanisms of pharmacoresistance in epilepsy, including the effect of genetic and molecular factors. In recent years, the increased knowledge generated by high‐throughput omic technologies has significantly improved the power of molecular genetic studies to discover new mechanisms leading to disease and response to treatment. In this review, we present and discuss the contribution of different omic modalities to understand the basic mechanisms determining pharmacoresistance in patients with MTLE+HS. We provide an overview and a critical discussion of the findings, limitations, new approaches, and future directions of these studies to improve the understanding of pharmacoresistance in MTLE+HS. However, it is important to point out that, as with other complex traits, pharmacoresistance to anti‐seizure medications is likely a multifactorial condition in which gene‐gene and gene‐environment interactions play an important role. Thus, studies using multidimensional approaches are more likely to unravel these intricate biological processes.

## 1 INTRODUCTION

Mesial temporal lobe epilepsy (MTLE) is the most common type of focal epilepsy in adult patients.^1^ Hippocampal sclerosis (HS) is a specific histopathological feature present in many patients with MTLE and can be detected by magnetic resonance imaging (MRI).^2^, ^3^, ^4^ Hippocampal sclerosis is characterized by neuronal cell loss and gliosis; it is classified into four histopathological types based on the distribution and extent of neuronal loss and gliosis in the *cornu ammonis* and the dentate gyrus of the hippocampus.^3^

Although many anti‐seizure medications (ASMs) are available for treating epilepsy,^5^, ^6^ at least one‐third of patients with MTLE+HS will be pharmacoresistant even with optimal clinical treatment.^4^ For these patients, epilepsy surgery is an option for better seizure control.^7^, ^8^ However, not all patients undergoing epilepsy surgery will be seizure‐free,^9^ underscoring the need for additional forms of treatment for these patients. In the search for new treatment options, it is undoubtedly relevant to investigate the mechanisms leading to pharmacoresistance, pointing to new treatment targets.

It is generally accepted that pharmacoresistance is a multifactorial process influenced by age,^10^, ^11^ different physiological states such as pregnancy,^12^ concomitant use of medications competing for the same metabolizing enzymes or drug transporters,^13^, ^14^ and the presence of comorbidities, including obesity,^15^, ^16^, ^17^, ^18^, ^19^ among others. Researchers have proposed different hypotheses to explain the mechanisms leading to pharmacoresistance in epilepsy, including (a) the “transporter hypothesis,” which proposes that pharmacoresistance results from the overexpression of multi‐drug efflux proteins, such as the ATP‐binding cassette (ABC) transporter superfamily, at the blood‐brain barrier, leading to abnormally lower levels of the ASM in the target^20^, ^21^, ^22^; (b) the “target hypothesis,” which postulates that the basic mechanisms leading to epilepsy are also involved in changes in the sensitivity of the drug targets; (c) the “neuronal network hypothesis,” which suggests that frequent excitatory stimulation of neurons, as seen in epilepsy, induces several biological processes that generate an anomalous neuronal network that leads to ASM resistance^23^; (d) the “intrinsic severity hypothesis,” which proposes that neurobiological factors may easily trigger severe and more constant seizures, leading to intractable epilepsy^24^; and (e) the “genetic variant hypothesis,” suggesting that variants in genes encoding drug‐metabolizing enzymes, drug transporters involved in the pharmacokinetics and pharmacodynamics of the ASM, and ASM targets, such as ion channels and receptors, could influence the pharmacologic response in patients with epilepsy.^25^

Our overall view is that all these hypothesized explanations likely play a role in pharmacoresistance in patients with epilepsy. Furthermore, we believe that studying the molecular mechanisms involved in pharmacoresistance could provide insights into the contribution of the different hypotheses. Thus, in this review we present and discuss the contribution of different modalities of molecular studies, especially high‐throughput molecular studies, also called omic technologies, to better understand the basic mechanisms involved in pharmacoresistance. Furthermore, because the etiology of epilepsy may influence pharmacoresistance, we focus on one specific epilepsy syndrome, MTLE+HS. We divide our text by omic modality, and within each modality, we review work on patients with epilepsy and animal models of MTLE+HS. We expect this work to provide an overview and critical discussion of the findings and limitations of these studies and propose new approaches and future directions.

## 2 METHODOLOGICAL PROCEDURE

Our searches of the literature were performed in MEDLINE via the PubMed database (http://www.ncbi.nlm.nih.gov) and Web of Knowledge. We looked for original studies, with the following keywords: mesial temporal lobe epilepsy, temporal lobe epilepsy, MTLE, or TLE combined with the following terms: “pharmacogen,*” “genomics,” “genetics,” “response to medication,” “refractoriness,” “pharmacoresistance,” “drug‐resistance,” “genome‐wide association study,” “epilepsy,” “transcriptomics,” “metabolomics,” “proteomics,” “hippocampal sclerosis,” “animal models,” “drug‐resistance epilepsy,” “gene expression,” “non‐coding RNA,” “ncRNA,” “micro‐RNA,” “miRNA,” “long non‐coding RNA,” “lncRNA,” “circRNA,” “DNA methylation,” “ketogenic diet,” “epigenetics,” “histone,” “biomarker,” “DNMT inhibitors,” “multi‐omics,” and “integration analysis.”

We also hand‐searched the reference lists of every primary study, previously published systematic reviews, and other types of review articles. Next, we carried out an extensive review of the references for relevant articles.

## 3 GENETICS

The genetic aspects influencing pharmacoresistance to ASMs are thought to affect the classic mechanisms in pharmacology, such as pharmacokinetics and pharmacodynamics (Table 1).^26^ As stated previously, one hypothesis to explain pharmacoresistance proposes that genetic variants impacting the function of candidate genes—such as those encoding drug‐metabolizing enzymes, drug transporters, or ASM targets such as ion channels and receptors—could influence the pharmacologic response in patients with epilepsy.^25^ Thus, we report the genetic studies addressing the different categories of candidate genes. Furthermore, although comprehensive genomic studies have been performed addressing the predisposition to epilepsy,^27^ we could not locate studies using agnostic genomic strategies to investigate the response to therapy in patients with epilepsy. Hence, all studies discussed here are based on the “candidate gene” strategy.

**TABLE 1:** Genetic variants putatively influencing the pharmacokinetics or the pharmacodynamics of anti‐seizure medications

Anti‐seizure medication	Metabolizing genes	Transporter genes	Pharmacodynamic genes	Target genes
Carbamazepine	*CYP1A2*, *CYP2A6*, *CYP2B6*, *CYP2C19*, *CYP2C8*, *CYP2E1*, *CYP3A4*, *CYP3A5*, *CYP3A7*, *NR1I2*, *NR1I3*, *EPHC1*, *MPO*, *UGT2B7*	*ABCB1*, *ABCC2*, *RALPBP1* (*RLIP76*)	*SCN1A*, *SCN1B*, *SCN2A*, *SCN3A*	Na^+^ channel
Phenytoin	*COMT*, *CYP1A2*, *CYP2A6*, *CYP2B6*, *CYP2C19*, *CYP2C8*, *CYP2C9*, *CYP2D6*, *CYP2E1*, *CYP3A4*, *CYP3A5*, *CYP3A7*, *EPHX1*, *UGT1A1*, *UGT1A4*, *UGT1A6*, *UGT1A9*, *NQO1*, *PTGIS*, *TBXAS1*	*ABCB1*	*SCN1A*, *SCN2A*, *SCN3A*	Na^+^ channel
Valproic acid	*UGT1A6*, *CYP2C9*, *CYP2A6*, *CYP2B6*, *ACADSB*, *ECH1*, *HSD17B10*, *IVD*			Na^+^ channel (?) and GABA transaminase inhibition
Lamotrigine	*CYP2A6*, *CYP2D6*, *ESR1*, *NR1I2*, *NR1I3*, *SLC22A1*, *UGT1A3*, *UGT1A4*, *UGT2B7*	*ABCB1*, *ABCC1*, *ABCC2*, *ABCC3*, *ABCC4*, *ABCG2*	*SCN1A*, *SCN2A*, *SCN3A*, *SCN8A*, *CACNA1*	Na^+^ and Ca^2+^ channels blocker
Benzodiazepines	*CYP1A2*, *CYP2C19*, *CYP2D6*, *CYP3A4*, *CYP3A5*, *NAT2*, *UGT1A4*, *UGT1A9*, *UGT2B15*, *UGT2B4*, *UGT2B7*		*ACBD3*, *DBI*, *GABRA1*, *GABRB2*, *GABRG2*, *SLC25A4*, *SLC32A1*, *SLC6A1*, *SLC6A11*, *SLC6A12*, *SLC6A13*, *TSPO*, *TSPOAP1*, *VDAC1*	GABA_A_ receptor and peripheral receptors
Phenobarbital	*CYP1A2*, *CYP2A6*, *CYP2D6*, *CYP2E1*, *CYP3A4*, *GSTM1*, *GSTP1*, *GSTT1*, *SULT1A1*, *SULT1A3*, *SULT1A4*, *SULT1E1*, *SULT2A1*, *UGT1A1*, *UGT1A6*, *UGT1A9*, *UGT2B15*	*ABCC2*, *ABCC3*, *ABCC4*, *ABCG2*	*SCN2A*	GABA_A_ receptor, Na^+^ receptor(?)
Levetiracetam			*SCN1A*	Binding to SV2A
Topiramate			*SCN2A*, *GRIK1*	Na^+^ and Ca^2+^ channels
Zonisamide	*CYP3A4*		*SCN2A*, *GRIK1*	Na^+^ and Ca^2+^ channels
Gabapentin		*SLC22A4*, *ABCB1*	*CACNA2D1*, *CACNA2D2*, *CACNA2D3*, *CACNA2D4*,	α‐2‐δ subunits of Ca^2+^ channels
Ethosuximide	*CYP3A4*, *CYP2E1*			Ca^2+^ channel
Vigabatrin				GABA transaminase

Note

(?), inconsistent data. This information has been adapted from Whirl‐Carrillo et al,^42^ Van der Weide et al,^43^ and Karnes et al.^44^

### 3.1 Drug transporters

One of the first studies investigating the role of genetic polymorphism in pharmacoresistance in epilepsy was conducted by Siddiqui et al.^28^ The authors tested the hypothesis that the variant C3435T (rs1045642) in the ATB‐binding cassette sub‐family B member 1 (*ABCB1*, also known as *MDR1* and P‐glycoprotein) transporter gene may influence response to treatment. They considered that some ASMs are substrates to this transporter^29^ and that previous studies showed that *ABCB1* is overexpressed in endothelial cells from epileptic brains.^21^ This study enrolled 200 patients with ASM‐resistant epilepsy and 115 patients with drug‐responsive epilepsy. The authors found that refractory patients were more likely to be “C” homozygous, and the “C” allele appeared significantly more in the drug‐resistant group of patients. However, the authors did not classify patients according to epilepsy syndrome or seizure type, and the classification of pharmacoresistance used was also unclear.^28^

A subsequent study evaluated three SNPs in the *ABCB1* gene: rs1128503 (C1236T), rs2032582 (G2677T), and rs1045642 (C3435T).^30^ The authors analyzed the possible effect of the CGC haplotype on pharmacoresistance in 210 patients with TLE who underwent presurgical evaluation, including patients with MTLE+HS and a group with so‐called cryptogenic TLE without HS. Based on the response to treatment and the monthly average seizure frequency under ASM treatment, patients were classified into three groups: A—two or fewer seizures per month; B—three to five seizures per month; or C—six or more seizures per month. They found an increased frequency of homozygous carriers of the CGC haplotype in patients with a higher frequency of seizures (A = 9%, B = 22%, and C = 32%). Furthermore, patients who were homozygous for the CGC haplotype had a 4.67‐times higher risk of belonging to group C than to group A. The authors also claimed that the difference in allele frequencies was more prominent in the MTLE+HS subgroup. Due to the small sample size, the authors did not perform a formal statistical analysis in the MTLE+HS subgroup.^30^ This study was considered a confirmation of the previous results reported by Siddiqui et al.^28^ However, another study analyzed SNP rs1045642 (C3435T) at the *ABCB1* gene in 175 patients with TLE, 134 patients with drug‐responsive TLE, and 41 patients with drug‐resistant TLE and found no difference in allele frequency,^31^ starting a long controversy over the role of *ABCB1* in pharmacoresistance in patients with epilepsy. It is noteworthy that none of these studies presented a formal calculation of statistical power to detect a genetic association, which is a significant limitation.

Over the following years, many additional studies investigating *ABCB1* polymorphisms in epilepsy were published, including recent meta‐analyses.^32^, ^33^, ^34^, ^35^, ^36^, ^37^, ^38^, ^39^ Although still controversial, it seems most likely that specific polymorphisms in *ABCB1* are not responsible for a significant influence in response to ASMs in epilepsy.

In 2014, researchers analyzed seven SNPs in the *ABCB1* gene: rs1128503 (C1236T), rs2032582 (G2677T), rs1045642 (C3435T), rs3213619 (T129C), rs2214102 (21G/A), rs1202168 (+139C/T), and rs1922242 (276T/A). The authors also analyzed three SNPs in the *ABCG2* gene: rs2231142 (Gln141Lys; missense), rs72552713 (Gln126Ter; stop gain), and rs2231137 (Val12Met; missense). The authors evaluated 259 patients with MTLE+HS, all drug‐resistant, and compared them to 201 patients with juvenile myoclonic epilepsy, all drug‐responsive, and found no association with the ASM response.^40^ However, the study presents an important limitation because if differences had been found, it would have been difficult to know whether they were due to treatment response or differences in the etiology of epilepsy.

More recently, in our work, described in more detail below, we investigated several SNPs in multiple candidate genes, including two drug transporters, *ABCB1* and *ABCC2*,^41^ in a cohort of 237 patients with MTLE and found no association with *ABCB1*. However, we showed that a SNP (rs2756104BB) in *ABCC2* contributes to a statistical model to predict patients who are refractory to ASM treatment. In addition, we found that the *ABCC2* transcript was overexpressed in hippocampal tissue from patients with refractory MTLE+HS.^41^

### 3.2 Metabolizing enzymes

It is well recognized that genetic polymorphisms could directly impact the metabolism of ASMs by influencing the function of drug‐metabolizing enzymes. In this way, functional SNPs have been found for *CYP2D6*, *CYP3A4*, *CYP3A5*, *CYP2C19*, and *CYP2C9*,^42^ all genes coding for ASM‐metabolizing enzymes. It is well‐established that the *CYP2C9* SNPs rs1799853(CYP2C9*2) and rs1057910 (CYP2C9*3) lead to low activity and nonfunctional alleles, respectively. Thus, individuals with these alleles could have decreased metabolism and clearance of phenytoin (PHT) and an increased risk of drug toxicity compared with patients with two normally functioning alleles (CYP2C9*1).^43^, ^44^

Recently, we studied 237 patients with MTLE, 75 of whom were responsive and 162 refractory to treatment with ASM. We showed that a prediction algorithm using 56 SNPs located at drug‐metabolizing genes and one drug transporter could predict patients more likely to be refractory to clinical treatment.^41^ HS was also included in the prediction model and further increased the accuracy of prediction. We used the criteria proposed by the International League Against Epilepsy (ILAE) to classify patients who are refractory to ASM.^45^ Overall, taking all variables into account, the model had an 81.8% accuracy of prediction.^41^ The variables that contributed most to the predictive power were HS and SNPs located in the drug‐metabolizing genes *CYP1A2*, *CYP2C19*, *CYP3A5*, *CYP1B1*, *CYP2E1*, and *CYP34A*, as well as in the drug transporter gene *ABCC2*. Furthermore, we clearly showed that the response to ASMs is a polygenic trait, with multiple genetic factors involved.^41^

### 3.3 Anti‐seizure medication targets

The role of polymorphisms in the α1‐subunit of the sodium channel (*SCN1A*) gene and response to treatment with ASM has also been investigated. Mutations in *SCN1A* have been found in patients with monogenic forms of epilepsy,^46^ and voltage‐gated sodium channels are targets for several ASMs, including carbamazepine (CBZ), PHT, and lamotrigine (LTG).^47^ In the first report, including patients with focal and generalized epilepsy, the researchers studied the influence of SNP rs3812718 (also known as IVS5‐91G>A) on CBZ response in 104 patients with drug‐resistant epilepsy and 117 patients with drug‐responsive epilepsy from Japan. Patients were defined as having a good response to ASM treatment if they were without any type of seizures for a minimum of 1 year after receiving ASMs and ASM‐resistant if they presented uncontrolled seizures over 1 year after three attempts with different ASMs. The authors found a 2.7‐fold increase in the risk for CBZ resistance in patients who were homozygous for the allele “A” of SNP rs3812718.^48^ A subsequent study corroborates these findings in 448 Han Chinese patients with focal seizures.^49^ After adjusting for possible confounding factors, the authors found that the “A” allele of SNP rs3812718 in *SCN1A* predicted the nonretention of CBZ treatment.^49^ More recently, a study evaluated the possible effect of two SNPs (rs2298771 and rs3812718) in the *SCN1A* gene and the efficacy of CBZ monotherapy for focal seizures in a large cohort of 628 patients.^50^ Efficacy was defined as the percentage decrease in the number of seizures after 12 months of treatment. Patients who were homozygous for the “A” allele in SNP rs2298771 presented greater efficacy (decrease of >75% of seizures) than “G” allele carriers.^50^ Furthermore, the authors did not find significant results for SNP rs3812718, contrasting with the two previous studies described above.^48^, ^49^

Another study evaluated the role of a variable number of tandem repeats (VNTR) of 17 base pairs (bp) in intron 2 of the serotonin transporter (5‐HTT) gene (*SLC6A4*) in response to treatment in 105 patients with MTLE+HS, namely 74 refractory and 31 responsive.^51^ The homozygous 12‐repeat allele carriers were more likely to not respond to drug treatment compared with the 10‐repeat allele carriers. Interestingly, the authors analyzed their data using two different criteria for ASM response^28^, ^30^ and obtained similar results (*P* = 0.04 and *P* = 0.01). The VNTR in intron 2 of the *SLC6A4* gene is functionally active, and homozygous carriers of the 12‐repeat showed increased messenger RNA (mRNA) expression.^52^ Thus, the authors suggest that refractory patients may express more 5‐HTT, leading to a lower synaptic serotonin concentration and, consequently, favoring a seizure‐prone environment. A subsequent study also investigated the same VNTR in intron 2 and another VNTR in the *SLC6A4* gene promoter region.^53^ This VNTR is a 44‐bp insertion/deletion, which results in either a short (S) allele or a long (L) allele that increases 5‐HTT expression.^54^ The study included 101 patients with MTLE, with or without HS, and the authors evaluated seizure frequency in the last 6 months. Patients were grouped according to genotype combinations: “high‐expression” genotypes of *SLC6A4* (L/L and 12/12), “mild expression” (L/L 10/10, L/L 10/12, S/S 12/12, and S/L 12/12), and “low expression” (no L/L and no 12/12).^55^ The results showed that patients with high‐expression genotypes had higher seizure frequency. Additionally, these patients had shorter seizure‐free periods.^53^

Variants in genes encoding the gamma‐aminobutyric acid (GABA) receptors have also been investigated. The first study investigated four SNPs in GABA_A_ receptor subunits: rs2279020 (*GABRA1*), rs3219151 (*GABRA6*), rs2229944 (GABRB2), and rs211037 (*GABRG2*) in 240 patients with MTLE+HS (all drug‐resistant) and 201 patients with juvenile myoclonic epilepsy (all drug‐responsive) and found no differences in the distribution of allele frequencies.^56^

### 3.4 Other candidates

Apolipoprotein E (ApoE) plays a role in metabolizing lipids and in maintaining neuronal membranes.^57^ ApoE was found to be increased in the hippocampi of the kainic acid (KA)‐induced rat model of MTLE.^58^ Next, a case‐control study evaluated two variants in the *APOE* gene: the ε4 allele and the −491A/T promoter variant in 194 patients with MTLE—119 refractory and 75 responsive to ASM treatment—from the Chinese Han population.^59^ They reported that HS was present in 61.3% of patients from the refractory group and 9.3% of the responsive group. The authors found no significant differences in the frequency of *APOE* ε4 and −491A/T alleles, genotypes, or haplotypes between two groups of patients.^59^

Vaults are organelles involved in intracellular vesicular and nucleocytoplasmic transport and have been reported to be involved in multi‐drug resistance in cancer.^60^ Moreover, overexpression of the major vault protein (MVP) has been observed in brain tissue of animal models^61^ and patients with MTLE+HS,^62^ frontal lobe epilepsy,^63^ and focal epilepsy associated with ganglioma.^64^ Balan et al^65^ investigated the hypothesis that SNPs in the *MVP* gene could influence pharmacoresistance to ASM. They evaluated three SNPs (rs4788187, rs3815824, and rs3815823) at the *MVP* gene in 220 patients with MTLE+HS, all drug‐resistant, compared with 201 patients with juvenile myoclonic epilepsy, all drug‐responsive. They found no association.^65^

Insulin receptors regulate several biological pathways in the central nervous system, such as the recruitment of GABA_A_ receptors, synaptic density, and dendritic growth.^66^, ^67^ Moreover, expression of the insulin receptor is increased in the anterior temporal neocortex of patients with refractory epilepsy.^68^ Thus, Che et al,^69^ considering the hypothesis that functional polymorphisms in the insulin receptor (*INSR*) gene, the insulin receptor substrate 1 (*IRS1*) gene, and the insulin receptor substrate 2 (*IRS2*) gene could be associated with the response to ASM in patients with epilepsy, evaluated 376 patients with TLE—201 refractory and 175 responsive. They studied three SNPs in Chinese patients: H1085H C>T in the *INSR* gene, G972R in the *IRS1* gene, and 1057G>A in the *IRS2* gene.^69^ They demonstrated that genotypic and allelic distribution for the H1085H polymorphism differed significantly between the refractory and responsive groups. Carriers of the “T” allele had a higher risk of drug resistance than patients who were homozygous for the “C” allele. Moreover, logistic regression analysis that combined the data from the three SNPs indicated that the presence of the genotype combination *INSR* CT+TT and *IRS2* GA+AA increased the risk of drug resistance. However, the results lost statistical significance after adjustment for age and sex distribution in the two groups of patients.^69^

## 4 TRANSCRIPTOMICS

A genome generates a transcriptome set, which reflects the broad pattern of gene expression. Gene expression analysis, including the profiling of cells and tissues, has allowed the mapping of molecules related to physiological and pathological mechanisms. However, the transcriptome is highly dynamic and is directly influenced by the stage of development, environmental conditions, variability among tissue samples, different organisms, and different experimental methods and platforms.^70^, ^71^ Moreover, with the introduction of new high‐throughput massive sequencing technologies in the past decade, we can now estimate the abundance of different types of RNA molecules, making it possible to identify and to analyze biological processes, signaling pathways, and prospective biomarkers.^71^

In epilepsy, transcriptomic studies usually employ tissue samples most affected by the disease. Thus, in MTLE+HS, brain samples obtained from epilepsy surgery are most frequently used.

### 4.1 Coding RNA

In 2011, researchers used human tissue to evaluate the transcriptomic profile of the CA3 and CA4 transition regions of patients with refractory MTLE+HS, either with febrile seizures (FS) or without febrile seizures (NFS).^72^ The authors used a co‐expression network analysis that discriminated the two phenotypes, MTLE+HS and FS or MTLE+HS and NFS. The FS phenotype was more related to glutamatergic signaling, and the NFS phenotype was more related to GABAergic pathways. However, they also found commonly altered genes, such as *SSTR1*, *MYT1L*, and *NELL1*. Moreover, the authors pointed to two genes with the potential to become a therapeutic target: *SSTR1*, a significant hub in the FS and NFS phenotypes, and *CHRM2*, which may have a role in epilepsy susceptibility.^72^ In this work, the authors extracted the CA3 region from fresh tissue directly in the surgery room, which helps preserve the RNA integrity; however, it is also challenging to extract the area of interest precisely without proper staining to allow for the correct identification of the structures.

Also in 2011, Mirza et al^73^ performed a meta‐analysis including 12 large‐scale expression microarray studies of mesial temporal lobe structures resected from patients with refractory MTLE. They found 22 genes differentially expressed in at least three studies: *GABRA5*, *NRGN*, *CCL2*, *GFAP*, *CPLX2*, *ENC1*, *HPCAL4*, *INHBA*, *PLCB1*, *PSD*, *SNAP25*, *STMN2*, *CAPN3*, *CD99*, *CDK2AP1*, *DYNLT1*, *OGG1*, *PABPC4*, *RDX*, *SPEC*, *TF*, and *ZFP36L1*. Gene Ontology (GO) enrichment analysis demonstrated that the most critical molecular functions related to pharmacoresistance were calcium transport and signaling, cytoskeletal function, and transporter activity. Biological processes involved were synaptic transmission and plasticity, regulation of the action potential, cellular cation homeostasis, and axonal and dendritic morphogenesis.^73^ Furthermore, the authors identified five ABC transporters that were upregulated and could potentially lead to pharmacoresistance,^74^ including *ABCC4*, which was previously studied as a candidate gene for pharmacoresistance in epilepsy in an expression study.^75^ Although sophisticated in terms of the integrative analysis used, this study is only as good as the data of the several original works included. Thus, the intrinsic limitations of microarray expression studies are essential to consider, such as the limited number of genes analyzed and the lower sensitivity and specificity compared with more recent technology. Hence, there was the need for extensive validation experiments, which were not always executed in the different studies.

A subsequent study explored the molecular mechanisms of pharmacoresistance to the ASM levetiracetam (LEV) transcriptomic profiling of epileptic brain integrated with variant analysis at the genomic level. The results showed a distinct transcriptomic profile in patients not responding to LEV.^76^ The most significant findings were related to synapse architecture and function (*GSK3β*, *SV2A*, *AP3M2*, *DNM1*, *AMPH*, *VAMP3*, and others), suggesting that the LEV resistance could be due to failures on the endocytic processes, thus limiting the entry of the drug in the synapses. The authors also identified a promoter SNP, namely the rs9305614 “G” allele, which correlates to an increased abundance of mRNA transcribed from the *PIGP* gene. PIGP protein is an important component of the Wnt‐signaling pathway, and the SNP rs9305614 “G” allele had an increased frequency in patients not responding to LEV,^76^ indicating a possible genetic association.

In addition to samples from patients with MTLE+HS, animal models have also been used to investigate the molecular mechanisms underlying epilepsy and ASM resistance. Recently, a study using CRISPR technology increased the endogenous expression of the *Kcna1* gene in glutamatergic neurons to treat chemoconvulsant‐induced TLE.^77^ This gene encodes the Kv1.1 potassium channel, and it is necessary for the proper regulation of synaptic transmission and action potential firing.^78^ It is known that overexpression of *Kcna1* decreases neuronal excitability and neurotransmitter release and can suppress seizures in animal models.^79^, ^80^ In summary, the researchers found that upregulation of *Kcna1* decreased the maximal firing frequency and that neurons expressing *Kcna1* compared with controls fired less when exposed to the same synaptic input. Interestingly, they showed that the Kcna1‐dCas9A construct had no adverse effect in the mouse hippocampi and rescued the hippocampal functions impaired due to epilepsy. These findings suggest a possible new anti‐seizure gene therapy. The transcriptomic data were used to generate the GO enrichment analysis, revealing alterations in pathways of neurodegeneration and apoptosis, upregulation of genes implicated in neuronal activity, and reestablishing the normal expression of genes related to glutamatergic transmission and synapse function. Thus, the authors suggested that CRISPR‐mediated gene expression could be a new candidate approach for gene therapy for intractable TLE.^77^ Despite the study not being related explicitly to pharmacoresistance, the authors showed that new therapeutic strategies should be evaluated, such as CRISPR‐mediated gene expression. Moreover, they demonstrated how important the *Kcna1* gene is in the epilepsy context and highlighted that it is a good target for further investigation.

### 4.2 Noncoding RNA

Although noncoding RNAs (ncRNAs) do not encode proteins, they play a crucial role in regulating gene expression.^81^ ncRNA, both small (eg, microRNA [miRNA]) and long (lncRNA), have been studied in the context of epilepsy and may play a significant role as possible therapeutic alternatives since they have highly selective targeting.^81^

The deregulation of miRNAs and lncRNAs has been extensively studied in the pharmacoresistance of different cancers.^82^, ^83^, ^84^ However, there is much that has to be explored regarding the role of ncRNAs in refractory epilepsy.^85^

#### 4.2.1 microRNA

To our knowledge, the first investigation of ncRNAs in MTLE was performed in 2010 by Aronica et al.^86^ The authors identified the differential expression of miR‐146a both in the hippocampal tissue of an animal model and in patients with MTLE+HS. The authors suggested the role of miR‐146a in modulating the inflammatory response in MTLE+HS and as a potential therapeutic target.^86^ Since then, most studies evaluating ncRNAs in MTLE+HS have been focused on the search for biomarkers of disease. Thus, we review below only a few studies addressing ncRNAs in the context of ASM resistance and as potential therapeutic targets in MTLE+HS.

Jimenez‐Mateos et al^87^ investigated the role of miR‐134 in TLE using animal models, brain tissue from patients with pharmacoresistant MTLE+HS, and cell culture. The authors reported that miR‐134 is upregulated in hippocampal tissue of the animal models and patients with MTLE+HS.^87^ Still, they suggested that silencing miR‐134 promotes inhibition of seizures in the animal model. Thus, the authors demonstrated for the first time that inhibiting a single miRNA could be effective in treating seizures in an animal model of MTLE.^87^ Subsequently, Omran et al^88^ studied the hippocampal tissue of children with MTLE and immature lithium‐pilocarpine‐induced rats. The authors showed that the differential expression of the pro‐inflammatory cytokine interleukin 1beta (IL‐1β) and miR‐146a were associated with inflammation in these tissues, which vary depending on the stage of the disease. Given these results, the authors suggested that IL‐1β and miR‐146a could be potential therapeutic targets for MTLE because anti‐inflammatory strategies in their animal model had effectively controlled seizures.^88^

Subsequent studies in animal models have shown remarkable heterogeneity in the type of ncRNAs identified. This could be related to the intrinsic limitations of animal studies, such as the use of different animal models, using distinct inducing agents, and leading to epilepsy by different mechanisms. Furthermore, this same variability could also be present in the mechanisms leading to pharmacoresistance in these animal models. In this context, Moon et al,^85^ faced with the difficult task of developing an animal model for refractory epilepsies, generated a putative pharmacoresistant mouse induced by pilocarpine. The authors used LEV and valproic acid (VPA) to select mice that were divided into two groups, responsive and refractory, according to their response to the ASMs. Of the 37 animals with spontaneously recurrent seizures, 23 showed an improvement in seizures in response to ASMs, but seven were refractory to at least one ASM tested. The authors used behavioral tests and miRNA evaluation by microarray and found that most differently expressed miRNAs were common to both groups of epileptic mice, and some also appeared in the control mice. Only four miRNAs (miR‐206, miR‐374, miR‐142‐5p, and miR‐468) were identified exclusively in the refractory epilepsy group.^85^ Although this study brings interesting results on the potential role of miRNAs in refractory epilepsies, there are important methodological limitations to consider. The number of animals used to assess differential expression is small, the authors used only microarray data, and no validation of the differentially expressed miRNAs was performed. Moreover, the authors used total RNA from brain tissue, making it challenging to identify miRNAs involved with the specific epileptogenic focus in the mesial temporal structures. Furthermore, response to ASMs was evaluated after the 7‐day period, which we consider too short to assess the response to ASMs.

In 2014, researchers evaluated the genome‐wide profile of miRNAs using a rat model of TLE induced by amygdalar stimulation.^89^ The authors used high‐throughput sequencing of RNA extracted from the total hippocampus of six animals, which developed spontaneous seizures after 2 months of electric stimulation. They identified six miRNAs (miR‐455‐3p, miR‐345‐3p, miR‐423‐3p, miR‐54, miR‐365‐5p, and miR‐296‐5p) as differentially expressed in epileptic animals compared with controls. Reverse transcriptase quantitative polymerase chain reaction (RT‐qPCR) validated the changes in these miRNAs, and interestingly, they are all related to neuronal apoptosis pathways.^89^ The authors also showed that the levels of miR‐423‐3p associated with the protein RISC were even higher in tissue from the epileptic rats. By contrast, the level of caspase‐6, a target regulated by miR‐423‐3p, was reduced, suggesting that miR‐423‐3p promotes a protective effect against neuronal apoptosis in these epileptic rats.^89^ It is important to note that, to our knowledge, this is the first study to use a hypothesis‐driven high‐throughput approach to study miRNAs in tissue from an animal model of TLE in the chronic stage of the disease.

In 2020, Korotkov et al^90^ analyzed miR‐132 in three experimental settings, hippocampal tissue of patients with MTLE+HS, a mouse model of MTLE, and human astrocyte cell culture. The authors used RT‐qPCR and in situ hybridization, showing increased miR‐132 levels in both humans and mice, mainly in glia. Additionally, the authors demonstrated that by transfecting miR‐132 in cell cultures, there was a downregulation of pro‐epileptogenic factors, suggesting that miR‐132 could be a potential therapeutic target.^90^

In addition to studies in brain tissue, researchers have investigated circulating miRNAs in blood, plasma, and serum. In this case, miRNAs are studied as potential biomarkers of disease or response to treatment.^91^ To our knowledge, the first study to investigate circulating miRNAs as biomarkers for refractory MTLE was published in 2016 by Li et al.^92^ Initially, the authors evaluated the miRNA expression profile using microarray‐based technology in brain tissue of patients with refractory MTLE compared with controls. Subsequently, they investigated the differentially expressed miRNAs in plasma samples. They used a series of different experiments: RT‐qPCR, luciferase assay, and validation in an independent cohort. They found that miR‐153 was downregulated in the plasma of patients with refractory MTLE compared with controls. The authors suggested that the downregulation of miR‐153 could contribute to increased expression of hypoxia‐inducible factor 1α (HIF‐1α), which could be involved in the mechanisms leading to refractory MTLE. In addition, miR‐153 might be a potential biomarker and therapeutic target.^92^ In 2018, the same group analyzed plasma and brain tissue in a larger cohort of patients with refractory MTLE and found similar results, supporting the role of miR‐153 as a potential biomarker and therapeutic target in MTLE.^93^

Shen et al^94^ investigated the expression of miR‐145‐5p in the plasma of 40 patients with refractory epilepsy, 11 with MTLE, compared with samples from 42 controls. The authors showed that miR‐145‐5p levels differed in plasma of patients with refractory epilepsy, suggesting that it could be used as a biomarker for refractory epilpesy.^94^

In 2017, our group published an investigation of three candidate miRNAs in the plasma of patients with MTLE and focal epilepsy due to focal cortical dysplasia.^95^ The work was performed in two phases; initially, we investigated the expression of miR‐23a, miR‐3, and miR‐134 in 27 patients—14 with MTLE—and 16 controls. A second cohort of 65 patients with MTLE—38 with refractory MTLE—was used for validation. Overall, we found and validated the differential expression of miR‐134 in plasma of patients with MTLE; however, this miRNA was not differentially expressed compared with patients with refractory or responsive MTLE. Thus, our findings suggest a role for miR‐134 as a circulating biomarker for MTLE, but not for response to ASMs.^95^

In a subsequent study published in 2020, the authors analyzed two miRNAs, miR‐146a and miR‐134, in the plasma of 162 patients with different types of focal epilepsy.^96^ They showed that both miRNAs were abnormally expressed in patients with MTLE+HS. Furthermore, they showed that miR‐146a and miR‐134 plasma levels could discriminate between patients with responsive and refractory epilepsy with a moderate degree of confidence.^96^ Thus, based on the studies above and the fact that miR‐134 seems to have a potent anti‐seizure effect,^97^ it seems reasonable to suggest that miR‐134 should be further investigated as a possible therapeutic target in epilepsy.

Hamamoto et al,^98^ aiming to study the role of miRNAs as potential therapeutic targets in refractory MTLE, assessed miR‐219, miR‐181b, and miR‐195. According to the authors, these molecules had been described as regulators of the excitatory and inhibitory neurotransmitter receptors in the CNS. These miRNAs were analyzed by RT‐qPCR in hippocampal and amygdalar tissue of 18 patients with refractory MTLE, compared with 12 autopsy controls. The authors found that only miR‐219 was differentially expressed.^98^

In 2020, Wang et al^99^ investigated miR‐124 in the phosphoinositide 3‐kinase (PI3K)/Akt signaling pathways of mice with refractory epilepsy. The authors noted that a high miR‐124 plasma level seemed to play a protective role during seizures, indicating that miR‐124 could be a potential therapeutic target.^99^

Very recently, a pilot study published by Benedittis et al^100^ evaluated six circulating miRNAs (miR‐146a, miR‐142, miR‐223, miR‐132, miR‐138, and miR‐298) previously identified as potential biomarkers for TLE or predictive biomarkers of pharmacoresistance. miRNA expression differences were measured in serum by RT‐qPCR in 27 patients (17 resistant and 10 responders) and 20 control subjects. There were differences in the levels of these miRNAs according to the patient group, and the authors suggested that miR‐142, miR‐146a, and miR‐223 are potential diagnostic biomarkers for TLE and that miR‐142 and miR‐223 might also be potential biomarkers of pharmacoresistance.^100^

#### 4.2.2 Long noncoding RNA

lncRNAs play crucial roles in several diseases, but their potential in MTLE is still untapped. This class of ncRNAs has already been associated with the prediction of good responses to anticancer drugs.^84^ Thus, Xie et al^101^ tested the hypothesis that KCNQ1OT1 lncRNA, previously shown to influence response to treatment in patients with cancer, could also be involved in ASM response in human brain cells in vitro. The cells were treated with phenobarbital (PHB), and KCNQ1OT1 lncRNA was assessed by microarray, RT‐qPCR, Western blotting, and many other assays. The results indicate that KCNQ1OT1 lncRNA contributes to resistance to ASM by recruiting additional molecular partners, such as the miR‐138‐5p/nuclear factor kappa‐light‐chain‐enhancer of activated B cells (NF‐κB)/ABCB1 axis.^101^

More recently, Cai et al^102^ assessed the levels of ILF3‐AS1 lncRNA in the hippocampus and serum of 23 patients with refractory MTLE and 18 control subjects and found that ILF3‐AS1 lncRNA was significantly upregulated in patients. In addition, they showed that overexpression of ILF3‐AS1 lncRNA increased levels of pro‐inflammatory matrix metalloproteinases (MMP2, MMP3, MMP9, and MMP14) and decreased the expression of miR‐212, a target regulated by ILF3‐AS1 lncRNA.^102^

Xiao et al^103^ conducted an interesting study evaluating the methylation profile of ncRNAs in patients with MTLE. The authors assessed the whole blood of 30 patients with refractory MTLE and 30 controls, showing differential methylation in both miRNAs and lncRNAs. Furthermore, according to the authors, the hypermethylation of lncRNAs was related to ion channel activity and drug metabolism and they suggested that these lncRNAs could potentially be involved in the mechanism leading to refractory MTLE.^103^

To our knowledge, the first work suggesting lncRNAs as a potential therapeutic target for epilepsy was carried out in 2015 by Lee et al^104^ in animal models of MTLE. The authors studied the expression of lncRNAs in two animal models of MTLE, one induced by pilocarpine and one induced by KA. lncRNAs were assessed by expression microarray, which identified 384 and 279 lncRNAs differentially expressed in the pilocarpine and KA models, respectively. Of these, the authors validated 54 lncRNAs in the pilocarpine model and 14 lncRNAs in the KA model, by showing a co‐regulation agreement between the lncRNA and the target. Furthermore, they found that these lncRNAs were associated with specific biological functions, morphogenesis, and neuron differentiation.^104^

Han et al^105^ evaluated the lncRNA expression profile in the hippocampal tissue of a rat model of MTLE induced by KA. They used expression microarray and validation by RT‐qPCR. They found that H19 lncRNA is upregulated in the latent period of the KA model. Furthermore, the authors showed that by increasing the expression of H19 lncRNA, there was status epilepticus (SE)‐induced neuronal apoptosis. By contrast, inhibition of H19 lncRNA had a protective effect on SE‐induced neuronal apoptosis. Thus, they proposed that H19 lncRNA could be used as a therapeutic agent to prevent epileptogenesis by reducing SE‐induced brain injury. These findings were corroborated by a subsequent study of the same group, using different experimental approaches in animal models. They also performed Western blotting in hippocampal tissues surgically removed from patients with MTLE.^106^

Jang et al^107^ also assessed the role of lncRNA by expression microarray in a mouse model of MTLE induced by pilocarpine. They found more than 100 lncRNAs differentially expressed in the hippocampus and more than 700 lncRNAs in the cortex of the pilocarpine animals. Moreover, according to the authors, these lncRNAs have been associated with acute inflammation, calcium ion regulation, remodeling of the extracellular matrix, and neuronal differentiation.^107^

#### 4.2.3 Circular RNA

Like miRNA and lncRNA, circular RNA (circRNA) participates in transcriptional and posttranscriptional gene regulation and has also been studied in differential response to therapy in patients with cancer.^108^ One of the first studies to evaluate circRNA in MTLE was published in 2018 by Li et al.^109^ They evaluated global expression and characteristics of circRNAs in the temporal cortex of 17 surgical samples obtained from patients with refractory MTLE and 17 controls. High‐throughput sequencing data showed expression of almost 80 000 circRNAs, with approximately 85% of these being new types of circRNA. However, only 442 circRNAs were differentially expressed when comparing patients and controls. Of these, eight circRNAs were validated by RT‐qPCR. The two relevant differentially expressed circRNAs reported were circ‐EFCAB2 and circ‐DROSHA. According to the authors, these could be involved in the mechanisms leading to refractory MTLE.^109^ In the same year, Gong et al^110^ analyzed the expression profile of circRNAs in the temporal cortex of 22 patients with refractory MTLE compared with 22 controls. The authors carried out various experiments such as microarray, RT‐qPCR, cell transfection, and Western blotting. They reported the presence of at least 586 circRNAs differentially expressed in tissue from patients with MTLE. Furthermore, they validated some of the circRNAs in the plasma samples of the patients, showing that circRNA‐0067835 was significantly downregulated in brain tissue and plasma of patients. They also found that there was a correlation between surgical outcome, assessed by the Engel scale, and levels of circRNA‐0067835. According to the authors, circRNA‐0067835 could be involved in promoting apoptosis and regulation of *FOXO3a* via miR‐155.^110^

In 2020, Gray et al^111^ investigated patterns of circRNAs and interactions with miRNAs in patients with refractory MTLE. In this study, the authors compared 24 brain tissue samples (seven from the neocortex and 17 from the hippocampus) from patients with MTLE with 14 postmortem brain samples (six from the temporal cortex and eight from the hippocampus). The high‐throughput sequencing data obtained identified 1515 circRNAs, but only nine circRNAs were differentially expressed in patients. The authors also investigated circRNA‐miRNA‐mRNA interactions and associated these interactive networks with functions in the transduction and transcription of signals for neurons in the hippocampus, implying a potential role in the pathogenesis of refractory MTLE. In this way, the differently expressed circRNAs associated with miRNAs could be considered new therapeutic targets.^111^ Of note, two additional studies have also investigated the role of the circRNA‐miRNA axis in cell cultures in the context of epilepsy.^112^, ^113^ These findings indicate the importance of these interactions and reinforce the need for additional studies to explore better the role of circRNA‐miRNA interplay in the mechanism of leading to pharmacoresistance in MTLE.

## 5 PROTEOMICS

Proteomic studies can provide information regarding protein abundance and dynamics, helping to understand the biological mechanisms of disease development, biomarkers, and drug discovery. Proteomics also allows us to analyze the posttranslational modifications (PTMs) that may occur in the proteins and gives us insight into the protein activity at a specific time point.^114^ Target tissues, such as the human or animal brain, are more suitable for proteomics because these are specific places where molecular changes are happening.^115^, ^116^, ^117^ Although many studies have applied proteomics in the context of epilepsy, as we reviewed recently,^118^ there is still a lack of information regarding the proteins linked to ASM pharmacoresistance in epilepsy.

In 2005, researchers reported a non‐ABC drug transporter mechanism related to pharmacoresistance to ASMs.^119^ The authors focused on the effects of differential expression of RLIP76 in epileptic brain tissue. The study showed that RLIP76 is upregulated in the blood‐brain barrier of epileptic tissue. Furthermore, according to the authors, RLIP76 actively participates in the transport of two classic ASMs, PHT and CBZ. By using RLIP76 knockout mice, they demonstrated that due to impaired drug extrusion mechanisms, decreased expression of RLIP76 is linked to high toxicity after administration of PHT. Their findings suggest a role for RLIP76 in ASM pharmacoresistance in addition to the traditional drug transporters such as MDR.^119^, ^120^

A study published in 2018 evaluated the proteomic profile of hippocampal tissue surgically removed from patients with medically refractory MTLE, compared with tissue from autopsy.^121^ The study used two‐dimensional gel electrophoresis followed by liquid chromatography coupled with tandem mass spectrometry (LC−MS/MS) to identify differentially expressed proteins. Among the main findings, the authors reported that glutathione S‐transferase P (GSTP1) was expressed only in hippocampal tissue of patients. According to the authors, this enzyme has been linked to the liver inactivation of ASMs. Thus, the abnormal expression of GSTP1 in hippocampal tissue from patients with refractory MTLE could be related to the mechanisms of pharmacoresistance.^121^, ^122^, ^123^

Yet another study investigated the mitochondrial proteome of a PHT‐resistant epileptic animal model, obtained by the amygdala‐kindling method.^124^ The researchers identified decreased voltage‐dependent anion channel 1 (VDAC1) and voltage‐dependent anion channel 2 (VDAC2) in the pharmacoresistant animals. These proteins are major outer‐membrane transporters and play an important role in energy production, as apoptosis mediators, and in metabolite trafficking. VDAC alterations can result in energetic failure and apoptosis; therefore, the authors suggest that these findings could be highly relevant to the mechanisms leading to PHT‐refractory epilepsy in these animal models.^124^

## 6 METABOLOMICS

Metabolomics is the study and analysis of an organism's metabolites that are present in a specific cell group, tissue, or biological fluid. Metabolomics has been widely applied to study disease mechanisms and to identify disease biomarkers or response to treatment.^125^, ^126^ Metabolomics is a highly efficient molecular modality that can identify specific metabolites or molecular pathways linked to disease, information that could be subsequently translated to the clinic.^127^, ^128^ Metabolomic studies have increased significantly over the past few years, mainly due to technological improvements; however, studies in epilepsy are still lacking.

We identified a single report published recently in which that authors aimed to study specific metabolites in an in vivo assessment of patients with MTLE.^129^ The authors used MRI and proton magnetic resonance spectroscopy (^1^H‐MRS) to measure total *N*‐acetylaspartate/total creatine (tNAA/tCr), myo‐inositol/tCr (mIns/ tCr), and glutamate/tCr (Glu/tCr) in patients with unilateral MTLE, either refractory or responsive, and healthy controls. The results revealed an association of the neuronal damage identified by MRI with the tNAA/tCr values. Moreover, patients with pharmacoresistant MTLE and left HS showed more widespread abnormalities than patients with negative MRI, patients responsive to ASM therapy, and patients with right HS. The authors also reported decreased Glu/tCr, which suggests tissue damage and seizure activity. Meanwhile, impaired mIns/tCr seemed to be related to pharmacoresistant epilepsy and left HS.^129^

## 7 EPIGENOMICS

Epigenetic modifications—DNA methylation, histone modifications, and ncRNAs—have been implicated in different drug treatment responses in several disorders.^130^, ^131^ Epigenetic mechanisms are critical players in regulating gene expression, and as such, they could be involved in mechanisms putatively involved in ASM pharmacoresistance.^130^ Furthermore, ASMs could change epigenetic marks, leading to changes in gene expression.^131^ We discuss how DNA methylation and histone modifications may influence ASM pharmacoresistance in epilepsy.^130^, ^132^, ^133^, ^134^

### 7.1 DNA methylation

In 2011, the “methylation hypothesis in epileptogenesis” emerged, in which researchers discussed the possible induction of DNA methylation by seizures occurring in patients with MTLE.^135^, ^136^ DNA methylation is carried out by DNA methyltransferases (DNMT1, DNMT3a, and DNMT3b). These enzymes are responsible for transferring methyl groups from *S*‐adenosylmethionine (SAM) on cytosine residues in the DNA. Most DNA methylation occurs in the so‐called CpG (5′‐cytosine‐phosphate‐guanine‐3′) context, and it results in a 5‐methylcytosine (5mC).^134^ By contrast, the active DNA demethylation process is catalyzed by ten‐eleven translocation (TET) enzymes, which convert methyl groups into hydroxymethyl groups (5‐hydroxymethylcytosine [5hmC]).^131^

In 2017, Long et al^137^ assessed DNA methylation patterns in blood samples of 30 patients with MTLE compared with 30 controls. The authors used the 450k methylation array and identified more than 200 differentially methylated sites in patients. Among these, there were sites in genes of the cytochrome P450 (CYP) protein superfamily—CYP3A4, CYP3A43, and CYP2C9—which are involved in ASM metabolism.^137^ These changes potentially affect the response to ASM treatment.

Recently, Unal et al^138^ suggested that the abnormal DNA methylation in the Na^+^‐K^+^‐Cl^−^ cotransporter 1 (NKCC1) promoter region may be linked to refractory MTLE. NKCC1 is a neuronal membrane protein essential to regulating the intracellular Cl^‐^ concentration; its acts alongside the GABAergic response in synaptic inhibition. Modification of NKCC1 function and gene expression was reported to be related to neurological disorders, including epilepsy, and could be a potential pharmacologic target to regulate intracellular Cl^‐^ concentrations.^138^ Furthermore, the authors found hypomethylation in the NKCC1 promoter in blood samples from patients with refractory MTLE, suggesting that low DNA methylation levels in the NKCC1 promoter region may be linked to pharmacoresistance in these patients.^138^

In another important study, Xiao et al^134^ searched for a signature of methylated CpGs in blood samples of 30 patients with MTLE—10 refractory and 20 responsive. The authors used a 450k methylation array and found 99 differentially methylated CpGs in patients with refractory MTLE. Furthermore, the authors used a second validation cohort, 17 patients with refractory MTLE compared with 14 patients with ASM responsive MLTE. They confirmed the presence of a different methylation signature in patients with refractory MTLE. Interestingly, they also observed that the differentially methylated CpGs mapped to genes involved in the DNA methylation pathway.^134^

It has been demonstrated that adenosine plays a role in seizure control by activating adenosine A1 receptors (A1R), which are coupled to the G_i_ protein. Thus, decreased adenosine kinase (ADK) could result in decreased adenosine levels, leading to increased seizures.^139^ Adenosine is also involved in DNA and histone methylation by regulation of DNMT function.^130^, ^140^ The ketogenic diet (KD) has been shown to increase adenosine levels,^141^ consequently reducing DNA methylation.^141^, ^142^ The high‐fat, low‐carbohydrate KD is also considered an alternative therapy for drug‐resistant epilepsy.^143^, ^144^ Thus, an interesting link between the use of KD for the treatment of medically refractory epilepsy and the modulation of methylation in DNA and histones has emerged.^143^, ^144^ In 2013, Kobow et al^132^ assessed changes in DNA methylation in a drug‐resistant pilocarpine‐induced animal model of epilepsy fed with the KD, pilocarpine+KD. The authors observed a significant reduction in the DNA methylation of the animals fed with the KD, pilocarpine+KD, compared with the predominantly hypermethylated pilocarpine animals.^132^ Furthermore, animals treated with the KD presented a delay in the establishment of chronic seizures.

Williams‐Karnesky et al^140^ investigated whether adenosine therapy could reduce the hypermethylation state observed in a KA model of MTLE. The authors observed reduced DNA methylation in the animals treated with adenosine, combined with decreased mossy fiber sprouting in the dentate gyrus of the KA‐treated animals. Subsequently, Lusardi et al^142^ showed that the KD restored adenosine levels in hippocampal tissues of epilepsy‐induced animal models treated with pentylenetetrazole (PTZ) kindling and pilocarpine. They also found a global DNA methylation reduction in hippocampal tissues of epileptic animals fed with the KD. Seizure frequency was suppressed in both epileptic animal models treated with the KD.^142^ These findings suggest that adenosine could be an alternative therapy for refractory epilepsy.^140^, ^142^

It is worth mentioning that some ASMs might influence DNA methylation and thus lead to changes in gene expression in the brain.^131^ As such, VPA can promote active demethylation regardless of DNA replication and, therefore, erase epigenetic marks.^145^ Furthermore, VPA use has been associated with reducing the 5hmC levels of mitochondrial DNA (mtDNA) and TET1 mRNA levels.^131^ These changes in DNA methylation could lead to changes in gene expression of important genes, such as in the case of reelin.^131^, ^146^ This extracellular matrix protein is crucial during hippocampal development and maintenance of laminar organization.^146^ Indeed, the use of VPA has been linked to changes in cell proliferation in the rat hippocampus.^147^ All these observations could indicate a possible role of VPA in inducing changes that may be linked to the progression of epilepsy or pharmacoresistance.

Epigenetic inhibitors are one of the candidates for novel pharmacologic intervention in refractory epilepsy. Indeed, an adenosine kinase inhibitor (AKI) has been proposed as a possible new ASM.^148^, ^149^ An AKI is an indirect epigenetic inhibitor, affecting adenosine levels and modulating DNA methylation. Sarcosine (also known as *N*‐methylglycine) is another potential ASM; it plays a role in balancing methyl radicals in the transmethylation process.^149^ Shen et al^150^ recently investigated the role of sarcosine treatment in a rat kindling model. DNA methylation was evaluated in the 5mC and 5hmC contexts via immunohistochemistry. The authors found that the 5mC levels were lower in the dentate gyrus of the rats receiving sarcosine treatment. Moreover, TET1 expression was upregulated. Based on these results, the authors suggested that by acting in the demethylation pathway, sarcosine could attenuate the progression of epilepsy.^150^

DNMTs also play a crucial role in DNA methylation, and changes in these enzymes have been observed in tissue from patients with MTLE.^151^, ^152^, ^153^ However, to our knowledge, there is no published study in which the authors aimed to investigate the pharmacologic response of the DNMT inhibitors and their possible relationship to pharmacoresistance in patients with MTLE.^130^

### 7.2 Histone modifications

Posttranslational modifications of histones influence the conformational state of chromatin and thus affect the regulation of gene expression.^154^ PTMs are an important epigenetic modification, and there are different mechanisms by which histone PTMs may occur. Still, most studies have focused on acetylation, methylation, and phosphorylation of histone proteins. Histone acetylation is associated with gene activation, and histone methylation is associated with gene repression.^154^, ^155^ Since almost 20 years ago, when the first report of histone PTMs associated with TLE^156^ was published, many researchers have reported a possible role of histone PTMs in patients and animal models of MTLE. However, only a few studies have addressed the manipulation of histone PTMs as possible therapeutic targets in epilepsy; these are reviewed below.

Histone deacetylases (HDAC) are enzymes that assist in gene regulation by modulating the acetylation and deacetylation of histones. They play a crucial role in different neurological functions, such as GABAergic function, synaptic plasticity, synaptogenesis, and developmental neural networks.^157^ VPA is also recognized as an HDAC inhibitor, promoting increased histone H3 acetylation in the brain.^130^, ^132^, ^158^ In addition to VPA, topiramate (TPM) has been shown to influence histone hyperacetylation.^157^ However, the significance of ASM‐mediated inhibition of HDACs in the context of pharmacoresistance is still not entirely understood.

Huang et al^156^ studied the pilocarpine rat model and observed that SE activates histone acetylation precisely at the brain‐derived neurotrophic factor (BDNF) promoter. They also showed that the glutamate 2 (GluR2) receptor and the BDNF promoter are deacetylated and hyperacetylated, respectively, after seizures. Furthermore, they reported that the deacetylation of histone H4 promotes a negative regulation of GluR2 levels in the CA3 region of the hippocampus of these animals. Because inhibition of HDACs prevented and quickly reversed the deacetylation of histones associated with GluR2, the authors suggested that such a mechanism could be targeted for neuroprotective therapies.^156^

Jagirdar et al^159^ evaluated changes in the expression of HDACs in the KA‐induced mouse model. The authors showed that changes in the pattern of HDAC expression may have therapeutic potential in these animals.^159^ A study published in the following year (2016) by the same group corroborates the initial findings in the KA and pilocarpine models.^160^ Furthermore, the authors showed that HDAC5 and HDAC9 were upregulated in the granular cell layers in the CA1 and CA3 sectors of the KA and corresponded to granular cell dispersion events in the chronic phase of the model. On the other hand, in the pilocarpine model, there was downregulation of HDAC5 and HDAC9 without the presence of granular cell dispersion. The results presented by these two studies indicate the potential of HDACs as therapeutic targets in epilepsy; however, it also indicates that the mechanisms involved in HDAC regulation in epilepsy are complex and need further clarification.^159^, ^160^

In 2018, Reddy et al^133^ evaluated possible therapeutic mechanisms of HDACs in MTLE using the hippocampus kindling model. Sodium butyrate, an HDAC inhibitor, was used daily for 2 weeks, and according to the authors, it could interrupt epileptogenesis. They also discussed that the treatment had no toxic or behavioral effects, indicating that HDACs could be therapeutic agents for the prevention of MTLE.^133^

## 8 MULTI‐OMIC INTEGRATION

An integrative analysis across multiple omic modalities has been increasingly applied and may give a broader view of the biological aspects related to different disease states. One of the first studies to apply this type of approach to investigate pharmacoresistance in epilepsy was performed in a mouse model of epilepsy.^161^ The authors investigated hippocampal tissue of 100 animals treated with pilocarpine and 100 controls (pilocarpine‐naïve) and analyzed gene expression by quantifying mRNA and protein levels. They identified co‐expression modules based on the differential expression between epileptic and healthy hippocampi. Furthermore, they established correlations of the module expression with seizure frequency. Then, using a predictive framework tool, they identified the tyrosine kinase receptor Csf1R as a potential therapeutic target. In addition, the authors assessed the effect of a known Csf1R inhibitor in pilocarpine‐ and KA‐induced models and an ex vivo organotypic hippocampal slice culture, showing that blocking Csf1R attenuates epilepsy seizures.^161^

A subsequent work using a multi‐omic approach aimed to study the potential role of miRNAs as therapeutic targets for MTLE.^162^ In this work, the authors investigated several animal models of MTLE, induced with pilocarpine, KA, or perforant path stimulation. The hippocampus of these animals was analyzed in different periods, and high‐throughput sequencing of miRNAs identified over 400 differentially expressed miRNAs for each model. Then, the authors searched for conserved miRNAs in humans and designed customized antisense oligonucleotides for the miRNA‐predicted targets. They tested these molecules in knockdown models and identified several targets leading to decreased seizures and signs of neuroprotection in the animals. By using an integrated analysis including RNA‐seq, proteomics, and predicted targets for miRNAs, they observed that the transforming growth factor beta (TGF‐β) signaling pathway was a common mechanism involved in seizure modification in all three animal models studied. In addition, the authors showed that inhibition of TGF‐β signaling blocked the anti‐seizure effects identified.^162^ Among the many miRNAs identified in this agnostic study, only two, miR‐132 and miR‐146a, were previously studied as potential therapeutic targets for epilepsy.^90^, ^96^

## 9 FINAL REMARKS

Although many studies have addressed the molecular mechanisms leading to pharmacoresistance in MTLE+HS, and considerable knowledge has been produced over the past few years, these studies also have significant limitations. Indeed, very few studies address the issue of noncompliance as a cause of pharmacoresistance.^163^, ^164^ In addition, there is evidence that the response to ASM therapy in a given patient may change over time; thus, a cross‐sectional study evaluating patients at a single time point could be misleading.^165^, ^166^ We also found a remarkable heterogeneity in the definition of pharmacoresistance among the many studies reviewed here, making it difficult to compare the results of different studies. To address this issue, the ILAE formulated a consensus definition of drug‐resistant epilepsy to facilitate clinical research and to improve patient care.^45^ Furthermore, the number of patients enrolled in these studies is still small; only a few studies recruited over 100 patients, and none of the studies reported here included more than 1000 patients. Finally, replication is still lacking for both clinical and preclinical studies.

Unfortunately, the initial progress in the genetic studies of pharmacoresistance in epilepsy was hampered by the controversy involving the putative association of a specific polymorphism in the *ABCB1* gene with pharmacologic response in patients with epilepsy,^30^, ^32^, ^33^, ^34^, ^35^, ^36^, ^37^, ^38^, ^167^ which recent larger studies and meta‐analyses have refuted.^39^, ^168^, ^169^, ^170^, ^171^ Our view on this matter is that even if there is an influence of *ABCB1* in pharmacoresistance, the effect is most likely small and insufficient to influence the phenotype alone.

Most pharmacogenetic studies in patients with MTLE+HS have focused on genetic variants in candidate genes, such as drug transporters, receptors, and metabolizing enzymes. A few other genes (*SLC6A4*, *APOE*, *INSR*, and *MVP*) have also been investigated, but the results have not been encouraging. However, most studies addressing the genetic determinants of pharmacoresistance only tested a single locus with few polymorphisms, which is a significant limitation, considering the likely polygenic nature of the phenotype (See Table 2 for a summary of the results). Moreover, except for one study,^41^ they all tested for a genetic association, which is fundamentally different from testing the predictive value of the genetic biomarker to identify patients most likely to be refractory to treatment with ASMs.^41^

**TABLE 2:** Main findings of studies exploring candidate gene for pharmacoresistance in mesial temporal lobe epilepsy

Year published	Genes studied (SNP/VNTR)	Main findings	Biological material	Organism	Method used	References
2003	*ABCB1* (3435C>T/rs1045642)	Refractory patients were more likely to be “C” homozygous, and the “C” allele appeared significantly more in the drug‐resistant group of patients	Blood samples	Human	Real‐time PCR and sequencing	^28^
2004	*ABCB1* (1236 C>T/rs1128503, 2677G>T/A/rs2032582, 3435C>T/rs1045642)	There was an increased frequency of homozygous carriers of the CGC haplotype in patients with a higher frequency of seizures, and homozygous carriers of the CGC haplotype had a higher risk of belonging to this group	Blood samples	Human	PCR	^30^
2005	*INSR* (H1085H C>T), *IRS1* (G972R), and *IRS2* (1057G>A)	Carriers of the “T” allele had a higher risk of drug resistance than patients who were homozygous for the “C” allele for SNP H1085H. Moreover, the presence of the genotype combination INSR CT +TT and IRS2 GA +AA increased the risk of drug resistance	Blood samples	Human	PCR‐RFLP	^69^
2008	*SCN1A* (IVS5‐91G>A/rs3812718)	Increase in the risk for CBZ resistance in patients who were homozygous for the allele “A”	Blood samples	Human	Real‐time PCR	^48^
2009	*SLC6A4* (VNTR intron 2)	Homozygous carriers of the 12‐repeat allele had a high risk of refractoriness	Blood samples	Human	PCR‐VNTR	^51^
2010	*SLC6A4* (VNTR in promoter region and intron 2)	Carriers of L/L and 12/12 genotypes had higher seizure frequency than patients in the other two groups	Blood samples	Human	PCR‐VNTR	^53^
2010	*APOE* (ε4, ‐491A>T)	No association	Blood samples	Human	PCR‐RFLP	^59^
2012	*SCN1A* (IVS5‐91G>A/rs3812718)	The “A” allele predicted the nonretention of CBZ treatment	Blood samples	Human	Sequencing	^49^
2013	*GABRA1* (rs2279020), *GABRA6* (rs3219151), *GABRB2* (rs2229944), and *GABRG2* (rs211037)	No association	Blood samples	Human	Real‐time PCR	^56^
2013	*MVP* (rs4788187, rs3815824, and rs3815823)	No association	Blood samples	Human	Real‐time PCR	^65^
2014	*ABCB1* (1236 C>T/rs1128503, 2677G>T/A/rs2032582, 3435C>T/rs1045642, 129 T>C/rs3213619, 21 G>A/rs2214102, 139 C>T/ rs1202168, 276 T>A/rs1922242) and *ABCG2* (rs2231142, rs72552713, rs2231137)	No association	Blood samples	Human	RFLP‐PCR, real time PCR, and sequencing	^40^
2014	*SCN1A* (rs2298771 and rs3812718)	People homozygous for the “A” allele in SNP rs2298771 presented greater efficacy (decrease of >75% of seizures) than “G” allele carriers	Blood samples	Human	Sequencing	^50^
2015	*ABCB1* (3435C>T/rs1045642)	No association	Blood samples	Human	Real‐time PCR	^31^
2017	*ABCB1*, *ABCC2*, *CYP1A1*, *CYP1A2*, *CYP1B1*, *CYP2C9*, *CYP2C19*, *CYP2D6*, *CYP2E1*, *CYP3A4*, and *CYP3A5* genes	56 SNPs located at drug metabolism genes (*CYP1A2*, *CYP2C19*, *CYP3A5*, *CYP1B1*, *CYP2E1*, and *CYP34A*) and one drug transporter (*ABCC2*) could predict patients more likely to be refractory to clinical treatment	Blood samples	Human	Multiplex‐PCR	^41^

Abbreviations: CBZ, carbamazepine; PCR, polymerase chain reaction; RFLP, restriction fragment length polymorphism; SNP, single nucleotide polymorphism; VNTR, variable number of tandem repeats.

The number of studies of different ncRNAs involved in pharmacoresistance and as potential therapeutic agents have been growing significantly over the past 5 years. However, there is a remarkable lack of consensus in the literature about the most relevant ncRNAs involved. As in the genetic studies, lack of reproducibility can be attributed to the different definitions of pharmacoresistance, small sample size, the technique used for identifying abnormally regulated ncRNAs, the use of different animal models, and the lack of validation of the results, among other factors (Table 3). However, it is clear that by studying gene expression (mRNA) and regulation (ncRNAs), additional insights into ASM pharmacoresistance can be obtained.

**TABLE 3:** Main findings of studies exploring transcriptomics in pharmacoresistance to anti‐seizure medication in mesial temporal lobe epilepsy

Year published	Transcript studied	Main findings	Biological material	Organism	Method used	References
2011	*SSTR1*, *MYT1L*, *NELL1*, and *CHRM2*	Molecular differences discriminated FS and NFS phenotypes. FS showed alterations more related to glutamatergic signaling and NFS to GABAergic pathways	Hippocampal tissue (CA3‐CA4)	Human	cDNA microarray	^72^
2011	*GABRA5*, *NRGN*, *CCL2*, *GFAP*, *CPLX2*, *ENC1*, *HPCAL4*, *INHBA*, *PLCB1*, *PSD*, *SNAP25*, *STMN2*, *CAPN3*, *CD99*, *CDK2AP1*, *DYNLT1*, *OGG1*, *PABPC4*, *RDX*, *SPEC*, *TF*, *ZFP36L1*, and *ABCC4*	Alterations in molecular functions such as calcium transport and signaling, cytoskeletal function, and transporter activity were the most relevant; synaptic transmission and plasticity, regulation of the action potential, cellular cation homeostasis, and axonal and dendritic morphogenesis were the most altered biological processes	Hippocampal tissue	Human	Meta‐analysis of microarray data	^73^
2013	*GSK3β*, *SV2A*, *AP3M2*, *DNM1*, *AMPH*, *VAMP3*, *PIGP*, and SNP rs9305614 “G” allele	The most significant findings were related to synapse architecture and function, suggesting that the LEV resistance may be due to failures on the endocytic processes, thus limiting the entry of the drug in the synapses. Moreover, the increased presence of the SNP rs9305614 “G” allele in patients resistant to LEV indicates a possible genetic association	Hippocampal tissue	Human	cDNA microarray	^76^
2020	*Kcna1*	Upregulation of *Kcna1* decreased the maximum firing frequency and neurons expressing *Kcna1* compared with controls fired less when exposed to the same synaptic input. Transcriptomic analysis revealed alterations in neurodegeneration and apoptosis pathways, upregulation of genes implicated in neuronal activity, and the reestablishment of normal expression of genes related to glutamatergic transmission and synapse function	Hippocampal tissue	Mouse	RNA‐seq, CRISPRa	^77^

Year published	Type of noncoding RNA studied	Main findings	Biological material	Organism	Method used	References
2010	miRNA	Differential expression of miR‐146a, with modulation of the inflammatory reaction; could be a potential therapeutic target	Hippocampal tissue	MTLE patients and rat electro‐stimulation model	RT‐qPCR and in situ hybridization	^86^
2012	miRNA	Differential expression of miR‐134; silencing this miRNA could be effective in treating seizures	Human temporal neocortex tissue and mouse hippocampal tissue	TLE patients and KA mouse model	RT‐qPCR, in situ hybridization, and Western blotting	^87^
2012	miRNA	Differential expression of IL‐1β and miR‐146a according to disease stage; both may be potential therapeutic targets for MTLE	Hippocampal tissue	Rat pilocarpine model and human children	RT‐qPCR	^88^
2014	miRNA	ASM response test: miR‐206, miR‐374, miR‐142‐5p, and miR‐468 as identified as differentially expressed in drug‐resistant	Brain tissue	Pilocarpine mouse model	Microarray	^85^
2014	miRNA	Differential expression of miR‐455‐3p, miR‐345‐3p, miR‐423‐3p, miR‐54, miR‐365‐5p, and miR‐296‐5p; miR‐423‐3p promotes a protective effect	Hippocampal tissue	Amygdala stimulation rat model	RNA‐seq, RT‐qPCR, Western blotting, and Nissl staining	^89^
2015	miRNA	miRNA‐134 has a protective effect on epileptic status	Hippocampal tissue	Pilocarpine mouse model	RT‐qPCR and Western blotting	^97^
2015	lncRNA	A speculative overview of lncRNAs as potential mechanisms for therapeutics	Brain tissue	KA and pilocarpine mouse model	Microarray	^104^
2016	miRNA	miR‐153 is involved in the mechanisms leading to refractory MTLE	Temporal cortex tissue and plasma	MTLE patients	Microarray, RT‐qPCR, and luciferase assay	^92^
2018	miRNA	miR‐153 as a potential biomarker and therapeutic target	Temporal cortex tissue and plasma	MTLE patients	RT‐qPCR, Western blotting, and luciferase assay	^93^
2018	miRNA and lncRNA	The methylation of ncRNAs could be used as potential biomarkers and therapeutic targets; hypermethylation of lncRNAs may be involved in drug‐resistant mechanisms	Whole blood	MTLE patients	Methylation array	^103^
2018	lncRNA	H19 had a protective effect on SE‐induced neuronal apoptosis; could be used as a therapeutic agent	Human and rat hippocampal tissues	TLE patients; KA and pilocarpine rat model	Microarray and RT‐qPCR	^105^
2018	lncRNA	lncRNAs are dysregulated according to the brain regions; lncRNAs are potential therapeutic targets	Hippocampal tissue	Pilocarpine mouse model	Microarray	^107^
2018	circRNA	circRNAs may be involved in the mechanisms leading to pharmacoresistance	Temporal cortex tissue	TLE patients	RNA‐seq and RT‐qPCR	^109^
2018	circRNA and miRNA	circRNA‐0067835 may be involved in promoting apoptosis and regulating FOXO3a via miR‐155113; could be a therapeutic target for TLE	Temporal cortex tissue and plasma	TLE patients	Microarray, RT‐qPCR, cell transfection, and Western blotting	^110^
2019	miRNA	miRNA‐145‐5p as a potential noninvasive biomarker for early detection of drug‐resistant epilepsy	Plasma	MTLE patients	RT‐qPCR	^94^
2019	lncRNA	lncRNA KCNQ1OT1 contributes to ASM pharmacoresistance	Human brain microvascular endothelial cells	Cell culture	Microarray, RT‐qPCR, Western blotting, and HPLC	^101^
2020	miRNA	miR‐132 overexpression; represents a potential therapeutic target	Human hippocampus tissue and rat brain tissue	TLE patients and electrical stimulation rat model	RT‐qPCR, in situ hybridization, and Western blotting	^90^
2020	miRNA	miR‐146a and miR‐134 plasma levels could discriminate between responsive and refractory epilepsy	Serum samples	TLE and focal epilepsy	RT‐qPCR	^96^
2020	miRNA	miR‐219 as a potential therapeutic target in refractory MTLE	Hippocampal and amygdalar tissues	MTLE patients	RT‐qPCR	^98^
2020	miRNA	miR‐124 may play a protective role during seizures, and it could be a therapeutic target	Plasma	Pilocarpine rat model	RT‐qPCR	^99^
2020	lncRNA	Overexpression of ILF3‐AS1; potential therapeutic target	Temporal cortex tissue and serum	MTLE patients	RT‐qPCR	^102^
2020	lncRNA and miRNA	Differently expressed circRNAs associated with miRNAs could be considered therapeutic targets	Temporal cortex and hippocampal tissue	MTLE patients	RNA‐seq	^111^
2021	miRNA	miR‐142 and miR‐223 as potential biomarkers associated with TLE pharmacoresistance	Serum	MTLE patients	RT‐qPCR	^100^
2021	circRNA and miRNA	circ_DROSHA as a potential prophylactic therapy against TLE progression	Serum specimens	MTLE cell model in vitro	RT‐qPCR, Western blotting, luciferase assay, and RNA immunoprecipitation assay	^113^

Abbreviations: ASM, anti‐seizure medication; cDNA, complementary DNA; circRNA, circular RNA; FS, febrile seizure; HPLC, high‐performance liquid chromatography; IL‐1β, interleukin 1beta; KA, kainic acid; LEV, levetiracetam; lncRNA, long noncoding RNA; miRNA, microRNA; MTLE, mesial temporal lobe epilepsy; ncRNA, noncoding RNA; NFS, nonfebrile seizure; RT‐qPCR: reverse transcriptase quantitative PCR; SE, status epilepticus.

Although many studies have applied proteomics to study the mechanisms underlying epilepsy, only a few of these have addressed the questions of ASM pharmacoresistance (Table 4). However, even with the few studies discussed here, several possible new treatment targets for epilepsy have been identified by proteomic studies. These need to be studied further so that the scientific community can adopt the most promising ones in preclinical studies.

**TABLE 4:** Main findings of proteomic studies in the investigation of pharmacoresistant mesial temporal lobe epilepsy

Year published	Proteins examined	Main findings	Biological material	Organism	Method used	References
2005	RalA‐binding protein 1 (RLIP76)	RLIP76 is upregulated in the blood‐brain barrier of epileptic tissue and actively participates in the transport of two classic ASMs, PHT and CBZ	Endothelial cells and glia from temporal lobe tissue	Human and mouse	Immunocytochemistry, cDNA, and microarray	^119^
2007	Voltage‐dependent anion channel 1 (VDAC1) and voltage‐dependent anion channel 2 (VDAC2)	VDAC alterations can result in energetic failure and apoptosis and may be highly relevant to the mechanisms leading to PHT‐refractory epilepsy in animal models	Hippocampal tissue	Rat	2‐DE–MALDI‐TOF	^124^
2018	Glutathione S‐transferase P (GSTP1)	GSTP1 has been linked to the liver inactivation of ASMs and was found expressed only in the hippocampus of patients with MTLE and could be related to the mechanisms of pharmacoresistance	Hippocampal tissue	Human	2‐DE–LC‐MS/MS	^121^

Abbreviations: 2‐DE, two‐dimensional gel electrophoresis; ASM, anti‐seizure medication; CBZ, carbamazepine; cDNA, complementary DNA; LC–MS/MS, liquid chromatography coupled to tandem mass spectrometry; MALDI‐TOF, matrix‐assisted laser desorption/ionization coupled with tandem time‐of‐flight mass spectrometry; MTLE, mesial temporal lobe epilepsy; PHT, phenytoin.

Despite the importance of evaluating the metabolic/metabolomic profile of pharmacoresistant epilepsy, there is a lack of studies analyzing metabolites and ASM responses (Table 5). Metabolomic studies may help better understand the bioavailability of several ASMs, specifically how they are absorbed, metabolized, and transported. In additional, metabolomics may help to unravel possible interactions of ASMs used in polytherapy and the effect of concomitant treatments with other drugs and food intake.^172^, ^173^ Remarkably, we could only identify a single in vivo study assessing metabolites in patients with refractory compared with responsive epilepsy. Thus, it is clear that this important field of investigation has a lot to offer in the future regarding the study of ASM pharmacoresistance in epilepsy.

**TABLE 5:** Main findings of metabolomic studies in the investigation of pharmacoresistant mesial temporal lobe epilepsy

Year published	Metabolites studied	Main findings	Biological material	Organism	Method used	References
2020	N‐acetylaspartate/total creatine (tNAA/tCr), myo‐inositol/tCr (mIns/ tCr), and glutamate/tCr (Glu/tCr)	There was an association between the neuronal damage identified by MRI with the tNAA/tCr values. There was decreased Glu/tCr, suggesting tissue damage and seizure activity. Meanwhile, impaired mIns/tCr seemed to be related to pharmacoresistant epilepsy and left HS.	In vivo image analysis	Human	MRI and H‐MRS	^129^

Abbreviations: H‐MRS, proton magnetic resonance spectroscopy; HS, hippocampal sclerosis; MRI, magnetic resonance imaging.

It is evident by the publications reviewed that the putative role of epigenetic mechanisms leading to ASM pharmacoresistance in epilepsy needs further exploration. So far, the epigenetic effects of ASMs have been explored in animal models of epilepsy (for a recent review on this subject, see Kobow and Blümcke^136^), but additional studies are also needed in patients. Of significant interest is that the KD may promote changes in DNA methylation, histone modifications, and ncRNAs,^174^ which should also be explored in further studies (Table 6).

**TABLE 6:** Main findings of epigenomic studies in the investigation of pharmacoresistant mesial temporal lobe epilepsy

Year published	Epigenetic marker	Main findings	Biological material	Organism	Method used	References
2013	DNA methylation	Lower DNA methylation levels in KA model of MTLE treated with adenosine	Hippocampal tissue	KA animal model	Methylation array and bisulfite sequencing	^140^
2013	DNA methylation	Hypermethylated status in pilocarpine animals and methylation reduction in pilocarpine+KD	Hippocampal tissue	Pilocarpine animal model	Sequencing of enriched methylated DNA	^132^
2015	DNA methylation	Lower genomic DNA methylation level in epileptic animals fed with the KD	Hippocampal tissue	PTZ kindling and pilocarpine animal models	5mC DNA ELISA	^142^
2017	DNA methylation	Several differentially methylated CpG sites in patients with MTLE are possibly related to cytochrome P450 protein superfamily	Blood samples	Patients and controls	Methylation array	^137^
2019	DNA methylation	Hypomethylation in the *NKCC1* promoter	Blood samples	Patients and controls	Methylation‐specific PCR	^138^
2020	DNA methylation	Several differentially methylated CpGs possibly correlated to drug resistance in patients with refractory MTLE	Blood samples	Patients, refractory or responsive	Methylation array	^134^
2020	DNA methylation	Reduction of 5mC levels in rats treated with sarcosine and upregulation of *TET1* gene expression	Hippocampal tissue	Rat model of rapid electrical hippocampal kindling	Immunohistochemistry and Western blotting	^150^
2002	Histone modifications	Histone acetylation in BDNF and GluR2; inhibition of HDACs as a potential mechanism for neuroprotective therapies	Hippocampal tissue	Pilocarpine rat model	ChIP assay	^156^
2015	Histone modifications	Changes in the expression levels of class I and IV HDACs in the epileptic hippocampus	Brain tissue	KA mouse model	In situ hybridization and Western blotting	^159^
2016	Histone modifications	Differences in the expression levels of HDAC5 and HDAC9	Brain tissue	KA and pilocarpine mouse models	In situ hybridization and Nissl staining	^160^
2018	Histone modifications	Sodium butyrate, an HDAC inhibitor, as a potential switch in epileptogenesis	Brain tissue	Classic and rapid hippocampal kindling mouse model	HDAC activity assay	^133^

Abbreviations: 5mc, 5‐methylcytosine; BDNF, brain‐derived neurotrophic factor; ChIP, chromatin immunoprecipitation; ELISA, enzyme‐linked immunosorbent assay; HDAC, histone deacetylase; KA, kainic acid; KD, ketogenic diet; PCR, polymerase chain reaction; PTZ, pentylenetetrazole.

Furthermore, other biomarkers, such as neuroimaging measurements, could constitute endophenotypes of great interest in studies about pharmacoresistance in epilpesy.^175^ Indeed, endophenotypes could be a more direct result of genetic influences, and by studying these intermediary characteristics one could better disentangle complex phenotypes such as pharmacoresistance to ASMs.^176^ An interesting prospective study aimed to identify early brain abnormalities that could precede pharmacoresistance in patients with MTLE has recently been published by Labate et al.^177^ The authors reported that before patients became refractory to ASM, they presented abnormalities in the white matter volume of the arcuate fasciculi, corticospinal tracts, left retrosplenial cingulum, and left inferior longitudinal fasciculus reduced. At follow‐up, these patients showed decreased fractional anisotropy in the corpus callosum, superior longitudinal fasciculi, and major bundles of the right hemisphere, independently of the presence of HS.^177^ In addition, a research protocol for a longitudinal study has been published, aiming to evaluate preexisting brain connectivity, molecular information, and the outcome of pharmacologic treatment in patients with newly diagnosed focal epilepsy.^178^

Finally, the contribution of integrating multi‐omic data in the study of pharmacoresistant MTLE is still unexplored^179^ (Table 7). As with other complex traits, pharmacoresistance to ASMs is likely a multifactorial condition in which gene‐gene and gene‐environment interactions play an essential role. Thus, studies using multidimensional approaches are more likely to unravel these intricate biological processes.

**TABLE 7:** Main findings of multi‐omics studies in the investigation of pharmacoresistant mesial temporal lobe epilepsy

Year published	Molecules	Main findings	Biological material	Organism	Method used	References
2018	mRNA, protein	Csf1R as a potential therapeutic target	Hippocampal tissue	Pilocarpine and KA mouse models and organotypic tissue culture	RNA‐seq, microarray, RT‐qPCR, in silico analysis, immunohistochemistry, and LDH assay	^161^
2020	miRNA, mRNA, and protein	Inhibition of TGF‐β signaling blocked the anti‐seizure effects. miR‐132 and miR‐146a could be potential therapeutic targets	Hippocampal tissue	Pilocarpine, KA, and PPS mouse models	miRNA‐seq, RNA‐seq, RT‐qPCR, and immunoprecipitation	^162^

Abbreviations: KA, kainic acid; LDH, lactate dehydrogenase; miRNA, microRNA; mRNA, messenger RNA; PPS, perforant path stimulation; RT‐qPCR, reverse transcriptase quantitative PCR; TGF‐β, transforming growth factor beta.

## 10 ETHICAL APPROVAL STATEMENT

We confirm that we have read the Journal's position on issues involved in ethical publication and affirm that this report is consistent with those guidelines.

## CONFLICT OF INTEREST

The authors declare that the research was conducted in the absence of any commercial or financial relationships that could be construed as a potential conflict of interest.
